# Reimagining teaching in higher education: Impact of a pilot physically active learning intervention on perceived teaching effectiveness

**DOI:** 10.3389/fspor.2026.1942048

**Published:** 2026-09-09

**Authors:** Beth McLeod, Samuel K. Lai, Jo Salmon, Natalie Lander

**Affiliations:** 1 School of Behavioural and Health Sciences, Faculty of Health Sciences, Australian Catholic University, Melbourne, VIC, Australia; 2 Deakin University Institute for Physical Activity and Nutrition, Burwood, VIC, Australia

**Keywords:** pedagody, physical activity, professional learning, teaching, university

## Abstract

**Purpose:**

Physically Active Learning (PAL) integrates purposeful movement into academic instruction and has been associated with improved student learning and health outcomes in school settings. However, evidence supporting PAL implementation in higher education is limited. This mixed-methods pilot study evaluated university educators' experiences of the TransformUs Higher Ed PAL program and explored perceived changes in teaching practice, competence, confidence, willingness, and PAL implementation.

**Methods:**

Educators from eight disciplines, including Law, Speech Pathology, Occupational Therapy, Nursing and Theology, at one Australian university participated in the intervention. Post-intervention semi-structured interviews (*n* = 10) were analysed using an inductive–deductive thematic approach informed by the Capability, Opportunity, Motivation–Behaviour (COM-B) model. Pre- and post-intervention surveys (*n* = 10) assessed perceived competence, confidence, willingness, barriers, and student outcomes associated with PAL, while implementation checklists (*n* = 6) described patterns of PAL implementation.

**Results:**

Educators reported increased psychological capability, enhanced motivation driven by improvements in student engagement, and highlighted the importance of practical, social, and institutional support for successful PAL implementation. Qualitative findings demonstrated that educators embedded PAL across diverse teaching contexts and described meaningful shifts in their teaching practices, student engagement, and professional identity. Survey findings indicated improvements across all five PAL implementation constructs, with educators reporting greater willingness, competence, confidence, and perceived student outcomes, alongside fewer perceived barriers to implementing PAL. Implementation checklists showed that educators consistently planned for PAL integration, while embedding PAL into assessment remained the most challenging aspect of implementation.

**Conclusions:**

TransformUs Higher Ed appears to be an acceptable professional learning approach for supporting PAL implementation in higher education. Although findings should be interpreted cautiously due to the small sample, they provide a strong rationale for larger multi-institutional studies incorporating observational and student-level outcomes to evaluate effectiveness, sustainability, and scalability.

## 1 Introduction

Teacher effectiveness is widely recognised as a critical determinant of student success, with a substantial body of research demonstrating a strong and consistent relationship between teacher quality and student achievement (1, 2). Among in-school factors, the quality of teaching has consistently shown to exert the greatest influence on student learning, surpassing the impact of curriculum design, school leadership, and material resources (3). Effective teaching is typically conceptualised as an interplay between teacher experience, subject knowledge, and pedagogical expertise, all of which contribute to student engagement, conceptual understanding, and academic outcomes (2, 4, 5). High-quality teaching extends beyond content mastery to include the use of responsive and inclusive instructional strategies that address diverse learner needs. Consequently, teacher quality, instructional effectiveness, and pedagogical practice are among the most powerful levers for improving educational outcomes (6).

One pedagogical approach that has demonstrated considerable promise in enhancing educational outcomes is Physically Active Learning (PAL). PAL integrates purposeful movement into academic instruction (7–11). A growing body of evidence highlights the positive impact of PAL on both academic outcomes (10, 12, 13) and on physical and mental health (9, 14, 15). The approach encompasses a range of strategies, including active breaks, embodied learning, and hands-on or experiential activities that align physical activity with curriculum content (16, 17). The theoretical foundations of PAL are informed by an evolutionary perspective of Embodied Cognition Theory (18), Self-Determination Theory (19), Constructivist Learning Theory (20), and Experiential Learning Theory (21), all of which emphasise the role of active engagement in knowledge construction. Through PAL, students become active co-constructors of knowledge, engaging physically, cognitively, and socially in the learning process (22, 23). Moreover, PAL supports a range of developmental domains including concentration, emotional regulation, impulse control, motivation, and classroom behaviour (24–26). In addition, PAL aligns with evidence-based teaching strategies such as collaborative learning, differentiation, scaffolding, worked examples, multiple exposures to content and metacognitive engagement. All approaches known to enhance educational outcomes across contexts (27, 28).

Given its documented benefits, research on PAL has expanded substantially in recent years (7, 8, 11). One notable example is TransformUs, a whole-school Australian initiative developed to reduce sedentary behaviour and increase physical activity through the integration of behavioural, environmental, and pedagogical strategies (29–31). While TransformUs has demonstrated effectiveness in supporting in-service teachers of children and adolescents to adopt more active teaching practices, sustained implementation in real-world classrooms remains challenged by time constraints, competing demands, and ingrained instructional habits (32, 33).

To address these challenges, a more sustainable solution involves embedding PAL principles into Initial Teacher Education (ITE). Exposure to active pedagogies early in professional preparation can foster pedagogical competence and confidence among pre-service teachers, and encourage the development of innovative, student-centred practices from the outset of their careers (34, 35). TransformUs Higher Ed was developed in response to the need for more systemic integration of active pedagogies in teacher preparation programs and equips pre-service teachers with practical strategies for embedding PAL into curriculum delivery (35–39). The program aligns closely with the Australian Professional Standards for Teachers at the Graduate level (40), supporting capabilities such as understanding learners, designing effective instruction, and fostering supportive learning environments. Findings of feasibility, effectiveness and implementation trials indicate that participation in TransformUs Higher Ed improves pre-service teachers' competence, confidence and motivation to integrate PAL strategies in their current and future teaching (35–39). Qualitative data further suggest that the program supports theory-to-practice transfer by helping pre-service teachers connect university coursework with classroom teaching demands (41).

Despite growing evidence of the effectiveness of PAL, its use in broader higher education settings beyond ITE, remains limited (42). University teaching continues to rely predominantly on didactic and sedentary models, offering few opportunities for embodied and active engagement (43, 44). Reported barriers to adoption in higher education include constraints such as lecture theatre design, limited awareness among academic staff, concerns regarding pedagogical legitimacy, and uncertainty about how to integrate movement meaningfully within the disciplinary content (43).

Effective teaching in higher education is widely recognised as multidimensional, requiring the ability to communicate complex ideas clearly, structure learning experiences coherently, foster inclusive environments, and engage students actively and meaningfully (45). The internationally recognised Professional Standards Framework (PSF) benchmarks success within teaching in higher education (46). The PSF outlines 15 dimensions of high-quality teaching which focus on evidence-informed, student-centred and impact-focus practices. The Australian University Teaching Criteria and Standards Framework identifies seven domains of quality teaching, including curriculum design, support for learning, assessment, learning environments, scholarly integration, evaluation of practice, and professional effectiveness (40). PAL is well aligned with these frameworks, supporting authentic, student-centred learning, collaboration, and the integration of theory with practice (47–50). Although research in primary and secondary education consistently demonstrates that PAL enhances student attention, engagement, academic performance, and learning satisfaction (8, 12), its uptake in higher education remains minimal (43, 49).

While TransformUs Higher Ed has demonstrated promising outcomes within ITE contexts, its impact on university teachers, or adult educators beyond ITE, has not yet been examined. There remains a pressing need for research exploring the applicability and impact of PAL within higher education teaching. This study seeks to address this gap by: (i) exploring university educators experiences and perceptions of PAL, including its perceived impact on teaching effectiveness and student outcomes; (ii) evaluating the impact of the TransformUs Higher Ed program on university teachers' competence, confidence, and willingness to integrate PAL into their teaching; and (iii) assessing the implementation of PAL strategies within their teaching practice.

## 2 Methods

### 2.1 Study design

This study used a mixed methods pilot design (51–53) to explore the impact of the TransformUs Higher Ed program on university teachers' perceived planning, competence, confidence, and implementation of PAL strategies, as well as the perceived effectiveness of their teaching practice. Where relevant, the CONSORT checklist for pilot trials informed the study design (53).

### 2.2 Participants and recruitment

Participants were recruited from one Australian university via digital advertisements distributed through institution email lists and intranet sites, as well as print advertisements displayed on campus noticeboards. Teaching staff eligible for inclusion were those delivering face-to-face classes in any degree, subject or program delivered at the university during the second half of 2025. Interested participants provided informed consent and completed a baseline survey accessed via an online link. Ethical approval was obtained from Deakin University's Human Ethics Advisory Group (HAE-20–170) and from the Australian Catholic University's Human Ethics Advisory Group (2025-4232E).

### 2.3 Intervention overview

The intervention was delivered over a 12-week academic semester, with support offered in the weeks before, during and after the semester. It was designed to support university educators to build teaching competency and integrate PAL strategies into their practice. The program followed a structured, five-phase model that aligned professional learning, implementation, observation and reflection to promote pedagogical change (37) (Supplementary File S1).

#### 2.3.1 Phase 1—program alignment and professional development workshop

The first phase involved strategic collaboration between the TransformUs Higher Ed research team and the university academic lead to align the program's pedagogical foundations with higher education teaching (adult learners), learning and teaching frameworks (i.e., PSF, The Australian University Teaching Criteria and Standards Framework) and institutional priorities (46). This alignment process ensured that PAL strategies were compatible with the university's curriculum goals, teaching frameworks, and staff evaluation and promotion models. A two-hour professional development workshop was subsequently co-developed and co-delivered by the TransformUs Higher Ed research team and the university academic lead, which served as the primary entry point for participating teaching staff. It introduced the pedagogical rationale for PAL, drawing on theoretical frameworks such as Embodied Cognition Theory and Constructivist Learning Theory, and presented evidence supporting the cognitive and behavioural benefits of integrating PAL into university teaching practice.

Participants experienced PAL strategies firsthand through a range of interactive activities and were provided with structured planning time to begin embedding these approaches within their own subject area. Emphasis was placed on alignment with the Australian University Teaching Criteria and Standards Framework [68], and other evidence-based models of teacher excellence in higher education (54). In addition, they were given guided access to the TransformUs online platform, which features a dedicated Higher Education professional learning section with various planning, implementation and reflective resources to support the implementation of PAL.

To maximise accessibility, additional repeat professional development sessions were delivered by the university lead for staff unable to attend the main workshop, ensuring broad engagement across disciplines and faculties.

#### 2.3.2 Phase 2—PAL implementation in teaching practice

Following the initial training, teachers were encouraged to integrate PAL strategies into their semester planning and teaching practice, across lectures, laboratories, seminars, workshops, and tutorials. These strategies included:

- Active breaks: short bouts of physical movement designed to energise and engage students in their learning;
- Active academic lessons: content-integrated movement tasks aligned with learning objectives to support conceptual understanding;
- Active learning environments: modifications to lecture theatre and classroom layouts (e.g., signage and desk configuration) to facilitate movement, interaction, and student engagement.

#### 2.3.3 Phase 3—observation and feedback on teaching practice

In the third phase, teaching staff were given the opportunity to have their teaching observed or their lesson plans reviewed by the research team. Following each observation or review, participants received formative feedback to support reflective discussion. These sessions highlighted strengths in implementing PAL, identified areas for improvement, and supported goal setting to enable deeper and more consistent integration of active teaching strategies.

#### 2.3.4 Phase 4—Mid-semester workshop

After approximately eight weeks of classes, the university teachers participated in a second 2-hour PAL workshop. This second workshop was designed to facilitate critical reflection, collaborative problem-solving, and continued professional development. The workshop served multiple purposes, including:

- Providing a supportive space for university teachers to share experiences and exchange practical insights related to PAL implementation.
- Facilitating discussion of implementation successes and barriers, informed by individual self-report adherence checklists completed during the semester.
- Encouraging deeper reflection on how TransformUs Higher Ed principles contributed to evolving teaching identity and teaching effectiveness.
- Offering tailored support, based on accumulated data from implementation checklists and observation feedback, with a particular focus on increasing pedagogical depth and expanding the use of PAL strategies.

#### 2.3.5 Phase 5—continued integration and support

In the final four weeks of the semester, university teachers were encouraged and supported to extend and consolidate their use of PAL strategies within their teaching practice. Ongoing mentorship supported participants to deepen the integration of PAL principles within their subject/s and to experiment with a broader range of active teaching approaches. University teachers were guided to make meaningful connections between PAL activities and student engagement, as well as to consolidate learning outcomes through embodied, collaborative, and active learning experiences. This final phase emphasised sustainability, positioning PAL not as an additional or peripheral strategy, but as a pedagogically sound, curriculum-aligned component of ongoing teaching practice in higher education.

### 2.4 Data collection

A multi-method data collection strategy was employed to explore the impact of the TransformUs Higher Ed intervention on university teachers' pedagogical practices and experiences. The primary method of data collection was a qualitative exploration of participants' experiences and perceptions. Following the intervention, all participating university teachers were invited to take part in individual semi-structured interviews, each lasting approximately 20–30 min. The lead author conducted all interviews. The purpose of these interviews was to generate rich, contextualised data to better understand the perceptions and experiences of each teacher's engagement with the TransformUs Higher Ed program and implementation of PAL within their practice. The Capability, Opportunity, Motivation–Behaviour (COM-B) model (55) was used to guide the development and structure of the interview protocol. This framework provided a theoretically informed lens through which to explore behavioural change processes and the factors influencing teachers' adoption or non-adoption of PAL strategies. Interview prompts were designed to elicit responses across the core COM-B domains: (i) psychological and physical capability (e.g., knowledge, confidence, skills, physical capacity); (ii) physical and social opportunity (e.g., institutional support, classroom environment, student engagement); and (iii) reflective and automatic motivation (e.g., pedagogical beliefs, professional identity, emotional influences). In addition, the interviews explored teachers' perceptions of the value and relevance of PAL within a higher education context, including its perceived impact on their own teaching practice and effectiveness, and on student outcomes (i.e., behaviour). Example interview questions included:

- Describe your experience of implementing PAL in your subject.
- What kinds of supports or challenges did you encounter when implementing PAL, and how did these influence your ability to integrate it into your teaching?

To enhance trustworthiness and credibility, member checking was employed during the interviews. Summaries of participants' responses were shared verbally throughout the interview process to verify interpretations, clarify meanings, and provide participants with the opportunity to confirm or amend representations of their perspectives (see Supplementary File S2).

Quantitative methods complemented the qualitative findings. Online surveys were administered at baseline and again 13 weeks later to measure self-reported changes in university teachers' perceptions of integrating PAL into their teaching. The surveys were adapted from validated instruments used in pervious TransformUs Higher Ed studies (35–39), and measured five key constructs: (i) willingness to integrate PAL into teaching practice; (ii) perceived impact of increased physical activity and reduced sedentary time on student learning outcomes; (iii) competence to implement specific PAL strategies in university classes; (iv) confidence to implement specific PAL strategies in university classes, and; (v) perceived barriers and facilitators to PAL implementation. These measures provided quantitative insights into the teachers' perceptions of their active pedagogical practices and enabled examination of attitudinal changes across the intervention period.

To assess program implementation, including fidelity and adherence to PAL strategies, university teachers completed a short online implementation checklist at weeks 4, 7, and 10 of the intervention. The checklist captured the frequency of PAL implementation using six items: planning, use of provided resources, integration within teaching, modelling of active pedagogy, provision of opportunities for student movement in classes, and integration within assessment. These self-reported data provided insights into temporal patterns and depth of PAL integration and informed the structure and content of the second workshop.

### 2.5 Data analysis

All interviews were transcribed verbatim using Microsoft Teams and were processed using Microsoft Word 2018 (Version 1806, Microsoft, Redmond, WA, USA) for text management. The analysis was guided by a hybrid inductive–deductive thematic approach, allowing for both data-driven insight and theoretical framing. The Capability, Opportunity, Motivation—Behaviour (COM-B) model (55) provided the analytical framework for deductive coding, enabling structured exploration of the behavioural mechanisms influencing PAL implementation. The analysis process was conducted by the first author and proceeded in the following stages (56):

- Familiarisation and initial inductive coding: Transcripts were read multiple times to ensure deep familiarity with the content. Basic editing was performed to enhance transcript readability. This involved removing filler words/sounds like “umm”, verbal brainstorming, such as “let me think” and repetition, like “yes absolutely, it was yes”. Manual open (inductive) coding was conducted to identify meaningful units of data.
- Thematic organisation: Initial codes were sorted and categorised according to the COM-B domains: *Capability* (psychological and physical), *Opportunity* (physical and social), *Motivation* (reflective and automatic) and *Behaviour*.
- Coding reliability: Consistent with a reflexive thematic analysis approach, formal inter-coder reliability was not sought. Coding was understood as an interpretative and reflexive process. To enhance analytic rigour, a subset of transcripts (i.e., three interviews) was discussed among the research team to support the development and refinement of codes and themes.
- Final coding and thematic refinement: The lead author then proceeded to code the remaining transcripts. The thematic structure was refined iteratively to ensure internal coherence within categories and to preserve fidelity to participants' narratives. Throughout the analysis, emergent themes were cross-checked against the original transcripts to confirm representativeness.

Quantitative data were analysed using descriptive statistics in Stata 18 (StataCorp, College Station, TX, USA). Given the small sample size, these results are presented as medians and interquartile ranges (IQRs). For the survey (see Supplementary File S3), each of the five PAL constructs comprised between 6 and 14 items rated on a five-point Likert scale (1 = strongly disagree to 5 = strongly agree). For participants who responded to all items within a construct, a construct-level summary score was calculated by summing all the items within that construct. Although individual Likert-scale items are ordinal, these multi-item summary scores were treated as approximately continuous, consistent with common practice for scales comprising five or more response categories. Median (IQR) construct scores are reported for each PAL construct.

The implementation checklist (see Supplementary File S4) consisted of six separate items assessing teachers' frequency of PAL use, each rated on a five-point scale (1 = never/no to 5 = as much as possible/yes). As each item represented a distinct component of PAL implementation, checklist items were analysed individually. Median (IQR) values are reported for each item, with missing responses excluded from the analysis of that item.

## 3 Results

### 3.1 Participant characteristics

Thirteen university teachers provided consent to take part in this study and 10 (Female=6) completed an interview (77% response rate) and survey, and six completed the checklists. Participants held a range of university roles, primarily lecturer and senior lecturer positions, and represented eight diverse disciplines including Education, Biomedical Science, Law, Nursing, Occupational Therapy, Speech Pathology, Theology and Public Health.

### 3.2 Qualitative results

Interview data illustrate how the educators' capability, opportunity, and motivation interacted to influence the behaviour of embedding PAL strategies in university teaching. Findings are presented under each of the COM-B component, supported by illustrative university teacher quotes.

#### 3.2.1 Capability

##### 3.2.1.1 Psychological capability

All university teachers reported limited to no prior knowledge of PAL, often expressing uncertainty regarding both its conceptual meaning and practical application prior to engaging in the TransformUs Higher Ed program. For several participants, PAL was described as entirely unfamiliar before the initial workshop, highlighting a clear baseline gap in psychological capability:

“I don't think I'd ever heard of physically active learning before, so it was a completely new thing for me.” (P5)

Within this context, the workshops, particularly their interactive and applied components, were consistently identified as critical in building foundational knowledge and understanding. Participants emphasised that these sessions not only introduced PAL, but also demonstrated its relevance and applicability within higher education teaching contexts:

“I had no idea what PAL was, so the workshop was really helpful to get me started.” (P4)

As familiarity with PAL increased, educators described a gradual shift in confidence and willingness to engage with the approach. Importantly, initial uncertainty was not eliminated but instead reframed as part of an iterative and shared learning process with students. This openness appeared to facilitate experimentation and reduce perceived risk:

“I let the students know this may not work. Let's just play with it and make adjustments as we go.” (P3)

Confidence was further strengthened through repeated implementation and exposure to practical examples provided during the workshops. These experiences appeared to legitimise PAL within participants' teaching practice, with some educators describing the workshops as providing both reassurance and a sense of professional permission to innovate:

“Having that workshop at the beginning was almost like a remit or permission to try other activities… It provided a bit of authorisation.” (P8)

Despite these gains, several participants highlighted structural limitations within higher education, particularly the lack of formal pedagogical training among university staff. This context contributed to an ongoing need for clearer, more structured guidance when implementing PAL strategies:

“Many staff in higher education are not formally trained as teachers, so it is even more important to support them with approaches that engage students effectively.” (P10)

Accordingly, participants emphasised the value of step-by-step instructional support, particularly for those new to PAL. Such scaffolding was perceived as essential for building confidence and enabling successful delivery:

“…for those new to PAL, detailed instructions help us feel confident and helps us deliver it more easily.” (P1)

In addition to enhancing confidence, structured guidance was also seen as a mechanism for reducing the planning burden and cognitive load associated with adapting PAL strategies to discipline-specific contexts:

“I wanted more step-by-step guidance to reduce the time burden of planning activities.” (P6)

Collectively, these findings suggest that while initial exposure and experiential learning opportunities were effective in building psychological capability, sustained confidence and implementation were further supported by the provision of clear, practical, and contextually relevant guidance.

##### 3.2.1.2 Physical capability

No participants reported personal physical limitations that impeded their ability to facilitate PAL within their teaching. This suggests that, at an individual level, educators generally perceived themselves as physically capable of implementing PAL strategies. However, considerations around students' physical capabilities emerged as an important factor influencing implementation. For example, one participant reflected on the need to better account for the diverse mobility levels of older adult learners within complex teaching environments:

“I don't think I did the best job of catering for their [older adults] speed and ability to move around a multi-tiered space.” (P2)

More broadly, participants demonstrated an awareness of the need to adapt PAL activities to accommodate both the physical and psychosocial needs of their students. Educators highlighted the importance of ensuring physical accessibility alongside creating a psychologically safe learning environment. Concerns around student self-consciousness—especially among younger cohorts—shaped the selection and design of activities:

“I avoided anything that required standing out physically … these 20-year-olds, they're so self-conscious and don't want to feel embarrassed. So, I avoided anything that might make them look foolish.” (P6)

In response, several participants described intentionally scaffolding PAL implementation to gradually increase student comfort and participation. This progressive approach was seen as critical for fostering a sense of safety and inclusivity:

“I felt it was important to ramp up gradually to create a sense of safety.” (P3)

Taken together, while educators did not perceive their own physical capability as a barrier, their accounts highlight the importance of adapting PAL to align with the varied physical abilities and psychological readiness of students, underscoring the interplay between physical and social considerations in effective implementation.

#### 3.2.2 Opportunity

##### 3.2.2.1 Physical opportunity

University teachers consistently identified time, space, and resources as key environmental factors influencing their capacity to embed PAL within their teaching. Among these, time constraints associated with planning and preparation emerged as the most prominent barrier, often situated within the broader context of competing academic demands:

“The challenge was really just about the time… given all the competing demands.” (P6)

This perceived lack of time was further compounded by constraints within the physical teaching environment. In particular, participants described how classroom layouts, especially those characterised by fixed furniture or specialised equipment, limited opportunities for movement and restricted the feasibility of implementing PAL strategies:

“Definitely a big one is space… rooms cluttered with tables made movement difficult.” (P8)

In addition to time and space, access to and suitability of resources influenced implementation. While tangible materials such as balls, posters, and activity cards were generally viewed as useful in supporting PAL delivery, some participants perceived existing resources as misaligned with the higher education context. Specifically, resources were often seen as tailored to school-based settings, requiring considerable adaptation and, consequently, additional time investment:

“I didn't end up looking at [the resources] because I had this idea that it was kind of very high school focused.” (P8)

Collectively, these findings highlight how multiple dimensions of the physical environment—temporal, spatial, and material—interact to shape university teachers' opportunity to implement PAL, often reinforcing one another to constrain uptake within higher education contexts.

##### 3.2.2.2 Social opportunity

Collegial support emerged as a salient enabler of PAL implementation, with participants consistently highlighting the importance of encouragement, feedback, and a non-judgemental environment provided by both the project team and peers. This supportive climate appeared to foster confidence and reduce perceived risk when trialling new pedagogical approaches:

“I just thought the team were awesome… You were never critical, never judgmental.” (P6)

Beyond immediate project support, several educators situated their experiences within the broader context of higher education teaching, which was often described as inherently isolating. In this light, participants expressed a strong desire for more sustained, collaborative structures, such as communities of practice, to facilitate ongoing knowledge sharing, peer learning, and the continued development of PAL strategies:

“It would be great to have a community of practice to share ideas.” (P3)

At a broader institutional level, alignment with organisational structures and priorities was identified as critical for the long-term sustainability of PAL. Participants suggested that embedding PAL within formal professional development pathways could normalise its use and support its integration into routine teaching practice. For example, one educator proposed its inclusion within accredited teaching qualifications to promote early adoption and cultural integration:

“I think it would be great if PAL was embedded in the Graduate Certificate in Higher Education. That way, new lecturers would learn these strategies early and it would become part of the culture rather than an optional extra.” (P2)

Taken together, these findings underscore the importance of social and institutional support systems in shaping opportunity, highlighting how interpersonal, community, and organisational factors collectively influence the uptake and sustainability of PAL in higher education contexts.

#### 3.2.3 Motivation

##### 3.2.3.1 Reflective motivation

Participants' motivation to implement PAL was strongly underpinned by a desire to enhance student engagement, particularly within teaching contexts where attention, attendance, or participation were perceived to be low. In this sense, PAL was viewed as a practical strategy to shift learning from passive to more interactive forms of engagement:

“I wanted to make my classes more interactive and less passive.” (P1)

Beyond this, PAL was frequently described as aligning with educators' broader teaching philosophies and professional identities. Participants characterised themselves as reflective practitioners who were open to innovation and motivated to expand their pedagogical repertoire, positioning PAL as a natural extension of this orientation:

“Because I always like to find new tools to add to my toolbox, and I thought that was a good opportunity.” (P8)

This alignment appeared to extend beyond instructional practice to also influence educators' own affective experiences of teaching. Several participants reported that delivering PAL not only benefited students but also enhanced their own sense of motivation, enjoyment, and professional fulfilment, thereby reinforcing ongoing engagement with the approach:

“It supports me as a teacher too. I feel more motivated, inspired and energised when I deliver engaging classes in this way, which strengthens my passion for my work and my desire to do more.” (P10)

In addition to these intrinsic drivers, participants articulated strong beliefs regarding the pedagogical value of PAL for student learning. Specifically, PAL was perceived to support deeper engagement with content, improved knowledge retention, and, ultimately, better academic outcomes and persistence:

“This approach reinforces our goal for students to actively engage in sessions rather than simply receive information. We want them to participate, interact with the content, and build their own understanding. When students do this, they are more likely to retain knowledge, perform better in their classes, and ultimately remain at university.” (P4)

Collectively, these findings suggest that both intrinsic (e.g., professional identity, enjoyment) and outcome-based (e.g., perceived student benefits) motivational drivers played a central role in shaping educators' engagement with PAL, reinforcing its perceived value within higher education teaching practice.

##### 3.2.3.2 Automatic motivation

Emotional responses emerged as an important influence on the adoption of PAL, functioning as both a barrier and an enabler at different stages of implementation. In the early phases, participants commonly reported feelings of apprehension, particularly in relation to how students might perceive PAL activities. These concerns often centred on the potential for activities to be viewed as trivial or inappropriate within a higher education context:

“I worried students might think it was silly, but they enjoyed it.” (P3)

However, these initial anxieties were frequently mitigated through direct experience and student feedback. Positive student responses appeared to play a critical role in reshaping educators' emotional reactions, reinforcing the acceptability and value of PAL within their teaching:

“They said ‘it was fun'. And I think it's three words that have a lot of value because you don't usually leave a [discipline] classroom saying that was fun.” (P1)

Over time, such affirming experiences not only reduced apprehension but also contributed to more positive affective responses toward teaching. Participants described a sense of renewed enthusiasm and enjoyment associated with incorporating PAL, suggesting that emotional rewards further supported sustained engagement with the approach:

“PAL gave me renewed enthusiasm for teaching.” (P6)

Taken together, these findings highlight the dynamic role of emotion in shaping behaviour, with initial uncertainty giving way to positive reinforcement and increased motivation as educators observed favourable student outcomes and experienced enhanced teaching satisfaction.

#### 3.2.4 Behaviour

The target behaviour, embedding PAL strategies into teaching practice, was evidenced through participants' self-reported changes across a range of educational contexts. Educators described integrating PAL within lectures, tutorials, staff meetings, and professional learning sessions, indicating both the adaptability of the approach and its application beyond traditional classroom settings.

These reported changes in teaching practice were accompanied by perceived shifts in student behaviour. Participants consistently observed increases in student alertness, participation, and interaction following the introduction of PAL activities. Notably, three of the ten interviewees explicitly highlighted improvements in students' attentiveness, describing a visible transition from passive to active engagement during class:

“They woke up… They were willing to participate… They wanted to get things done to win.” (P9)

Such observations appeared to reinforce the perceived effectiveness of PAL, contributing to participants' intentions to sustain and further develop its use. All educators expressed a commitment to continuing PAL implementation in future teaching, often linking this intention to their beliefs about its positive impact on student engagement and learning outcomes:

“I'll definitely use it in future semesters because I honestly believe that it enhances engagement and learning.” (P7)

In addition to individual intentions, some participants positioned themselves as advocates for broader uptake, emphasising the potential for PAL to be embedded more widely within higher education practice. This included a desire to model effective implementation and contribute to cultural change at the faculty level:

“There is a clear place for it in our teaching practice, and I want to be an exemplar of how it can be implemented more broadly within the faculty.” (P3)

Collectively, these findings provide evidence of behaviour change, demonstrating not only the adoption of PAL in practice but also strong intentions for its sustained and expanded use, supported by perceived positive impacts on both teaching and student engagement.

### 3.3 Quantitative results

Ten participants completed the survey at both timepoints. All five pre/post survey construct summary scores changed between T1 and T2 (see Table 1). Median scores increased for Willingness (from 25.5 to 27.5), Student outcomes (from 28 to 32.5), Competence (from 22 to 29), and Confidence (from 16 to 26). The median Barriers score decreased from 37 to 27, suggesting fewer perceived challenges to implementing PAL. The largest changes were observed for Confidence and Barriers, with the median Confidence score increasing by 10 points and the median Barriers score decreasing by 10 points.

**Table 1:** Differences between timepoints for five constructs of PAL integration assessed in survey (*n* = 10).

PAL construct	T1 median (IQR)	T2 median (IQR)	Potential range
1. Willingness	25.5 (23; 28)	27.5 (26; 29)	6–30
2. Student outcomes	28 (26; 31)	32.5 (29; 33)	7–35
3. Competence	22 (17; 25)	29 (26; 32)	8–40
4. Confidence	16 (11; 20)	26 (23; 30)	8–40
5. Barriers	37 (30; 41)	27 (20; 31)	14–70

IQR, interquartile range.

During the intervention, participants completed three short implementation checklists, each comprising six questions. Five participants completed the week 4 checklist, four completed the week 7 checklist and three completed the week 10 checklist. Overall, six participants completed at least one checklist. Planning for PAL generally had one of the highest median score across the three checklists (5, 4, and 5) whilst Assessment consistently had the lowest median score (1, 1, and 1) (see Table 2).

**Table 2:** Difference between checklists for six PAL implementation items.

	Week 4 checklist median (IQR) (*n* = 5)	Week 7 checklist median (IQR) (*n* = 4)	Week 10 checklist median (IQR) (*n* = 3)	Potential range
1. Planned	5 (4; 5)	4 (3.5; 4.5)	5 (3; 5)	1–5
2. Resources	2 (2; 4)	3 (1.5; 4.5)	5 (4; 5)	1–5
3. Integrated	5 (5; 5)	3.5 (2; 4)	3 (3; 5)	1–5
4. Modelled	3 (3; 5)	2.5 (1.5; 3.5)	3 (3: 5)	1–5
5. Opportunities	4 (4; 5)	3 (2.5; 4)	4 (3: 5)	1–5
6. Assessment	1 (1; 2)	1 (1; 1)	1 (1; 3)	1–5

IQR, interquartile range.

## 4 Discussion

This pilot study sought to examine the perceived influence of the TransformUs Higher Ed intervention on teaching effectiveness and student outcomes, while also addressing a critical gap in the higher education literature by investigating its impact on university teachers' self-reported planning, competence, confidence, and implementation of PAL. Overall, the findings suggest that educators perceived the TransformUs Higher Ed program to support positive changes in their teaching practice and related behaviours. Qualitative data explained that educators actively implemented PAL across diverse teaching settings and experienced shifts in how they conceptualised teaching, engagement, and their professional role. Complementing these findings, quantitative results showed preliminary evidence of improvements in educators' competence, confidence, willingness to implement PAL, and perceived impact on student outcomes, alongside reductions in perceived barriers, indicating an enhanced readiness and capacity to embed PAL within higher education contexts.

Importantly, findings highlight that these changes were underpinned by interconnected behavioural mechanisms. In line with the COM-B model (55), increases in psychological capability were achieved through experiential workshops and practical demonstrations that built foundational knowledge, confidence, and skills. However, capability alone was insufficient to ensure implementation of PAL. The translation of capability into practice was influenced by opportunity-related factors, time constraints, teaching environments, and access to relevant resources, as well as collegial and institutional support. This aligns with systematic review evidence identifying ‘environmental context and resources' and 'social influences' as key determinants of translating evidence into practice (57). Motivation emerged as a critical reinforcing mechanism, with educators' intrinsic desire to enhance student engagement, alignment with professional identity, and positive emotional experiences, particularly in response to student feedback, supporting both initial adoption and sustained use. Congruent with previous research in teacher professional growth (37), these components operated synergistically, with supportive social environments and positive teaching experiences strengthening motivation, while observable improvements in student engagement reinforced confidence and continued implementation. Together, this dynamic interplay drove changes in teaching practice, highlighting the value of theoretically informed interventions combined with practice-based opportunities to explore, implement, and reflect (37).

The integration of qualitative and quantitative findings sheds light on the mechanisms driving these changes. For example, reductions in perceived barriers were explained by educators' accounts of how collegial support, leadership endorsement, and access to adaptable strategies helped them navigate structural constraints. This aligns with previous research emphasizing that collegiality, peer mentoring, and supportive teaching and learning cultures are crucial for the professional development of university teachers within their local disciplinary or departmental context (58). Although time and space remained persistent challenges, participants reported greater agency in managing these barriers, reflecting a shift not only in their presence but in how they were perceived and addressed. This change stemmed from increased knowledge and confidence, as well as shared strategies developed through a newly established community of practice and collaborative professional learning, which are known to enhance teaching in higher education (59). Motivational change was also evident across both data strands. Quantitative increases in willingness to implement PAL were reinforced qualitatively through educators' reflective beliefs about teaching effectiveness and automatic emotional responses, including enjoyment, renewed enthusiasm, and professional satisfaction, key drivers of teacher effectiveness (37, 60). Furthermore, student outcomes, or their responses to the integration of PAL by educators, appeared to drive changes in teacher motivation. This aligns with previous research indicating that classroom experiences demonstrating the impact of instructional strategies on student learning further reinforce their adoption. Student outcomes serve as a guiding principle for effective teaching, motivating educators to seek evidence-based strategies. In particular, positive student feedback, both verbal and behavioural, emerged as a powerful reinforcing mechanism, enhancing motivation and supporting the sustained use of PAL, while simultaneously fostering knowledge acquisition, skill development, and ongoing professional growth (37, 61).

These findings have important implications for professional development in higher education. Embedding PAL was not simply a matter of increasing awareness or willingness; it required structured opportunities to develop capability, supportive environments that enabled implementation, and reinforcing experiences that sustained motivation. Collegial support, collaboration, and the creation of communities of practice were particularly important, providing shared solutions, peer mentoring, and a sense of collective responsibility that facilitated the translation of knowledge into sustained teaching practice. Such social and collaborative mechanisms are recognized as key enablers of effective professional learning in higher education (58, 60). TransformUs Higher Ed was experienced not merely as training, but as an authorising structure that legitimised active pedagogies within a context where traditional, sedentary teaching practices often dominate (49). This sense of legitimacy was particularly important given that many higher education educators are appointed primarily for their disciplinary expertise rather than formal training in pedagogy. Consequently, although educators often possess deep subject-matter knowledge, limited pedagogical content knowledge can constrain their capacity to design and deliver effective learning experiences (62). This was especially relevant in the current study, which included educators from diverse disciplines such as Law, Education, Occupational Therapy, Speech Pathology, and Nursing, each characterised by specialised professional knowledge and discipline-specific approaches to teaching and learning. While disciplinary content and professional competencies necessarily differ, the findings suggest that the pedagogical principles underpinning PAL have the potential to enhance teaching practice across disciplines. These findings reinforce the need for professional learning that is practical, experiential, and contextually relevant, enabling educators to develop both pedagogical competence and confidence in authentic teaching contexts (37). Importantly, such professional learning should recognise disciplinary differences while also acknowledging the broader capacity of PAL to enhance teaching and student learning outcomes across diverse fields of study (63, 64).

However, the findings also highlight areas for further development. The lower implementation of assessment-related PAL strategies compared to other strategies suggests that while educators can readily integrate movement into teaching and learning activities, embedding PAL within assessment practices is more complex and may require additional conceptual and system-level support. This aligns with broader literature indicating that assessment is one of the most resistant domains of pedagogical change (65, 66). This may also reflect the fact that assessment structures and requirements are often determined at the program or institutional level, limiting individual educators' autonomy to modify assessment practices.

This pilot study makes several important contributions to higher education research. First, it extends the evidence base for PAL into the tertiary context, addressing a notable gap in a field that has predominantly focused on school-based settings. Second, it demonstrates the utility of the COM-B model as a framework for understanding pedagogical behaviour change in higher education. Third, it provides evidence that teaching effectiveness can be enhanced through structured, theory-informed professional learning interventions that support active pedagogies and foster collaborative, supportive environments for learning both content and how to teach it. By emphasizing behavioural mechanisms rather than outcomes alone, the study advances understanding of how sustainable changes in teaching practice can be achieved. Another key strength was the diversity of disciplines in which PAL was implemented. Eight fields, including Law, Occupational Therapy, Education, Nursing, and Speech Pathology, were represented, demonstrating that PAL is not confined to initial teacher education but has potential to enhance teaching and learning across a wide range of academic disciplines.

Several limitations should be considered when interpreting these findings. The sample size was small and drawn from a single institution, which may limit generalisability. Although positive changes were observed with the quantitative scores, these should be interpreted as indicative rather than definitive. The reliance on self-report measures also introduces the potential for social desirability bias, while incomplete implementation checklist responses may have resulted in non-response bias; however, the inclusion of qualitative data and implementation measures provides some degree of triangulation. Future research would benefit from a larger sample size, across multiple institutions. In addition, incorporating observational methods and longer-term follow-up to assess the sustainability of behaviour change would be beneficial. Finally, the study focused primarily on educator outcomes rather than direct measures of student learning. While university teachers reported positive student responses, future research should examine student-level outcomes more systematically to strengthen causal inferences.

## 5 Conclusion

This mixed-methods pilot study suggests that participating educators perceived TransformUs Higher Ed to effectively support the adoption of PAL within their teaching practice. Qualitative findings indicate that adoption was driven by enhanced capability through experiential learning, shaped by contextual opportunities such as time, space, and collegial support, and reinforced by strong motivational and emotional responses, particularly through positive student engagement. Quantitative data showed improvements in self-reported competence, confidence, willingness, and perceived student outcomes, alongside reduced barriers, suggesting increased readiness to implement PAL. Implementation checklists confirmed that PAL strategies were applied across diverse teaching contexts, with educators reporting higher student participation and clear intentions to sustain and expand their use. Collectively, these findings provide pilot evidence that structured, theory-informed PAL professional learning grounded in behaviour change principles can achieve meaningful pedagogical change in higher education. The results of this pilot study therefore strongly support the value of conducting a future definitive trial to rigorously evaluate the impact and scalability of TransformUs Higher Ed.

## Data Availability

The raw data supporting the conclusions of this article will be made available by the authors, without undue reservation.
