# Supplementary material for: Reimagining teaching in higher education: Impact of a pilot physically active learning intervention on perceived teaching effectiveness
Source: Front Sports Act Living. 2026 Sep 9;8:1942048. doi: 10.3389/fspor.2026.1942048 (PMC13597808; doi:10.3389/fspor.2026.1942048)
Supplement: Supplementary file 1 [file Supplementaryfile1.docx]

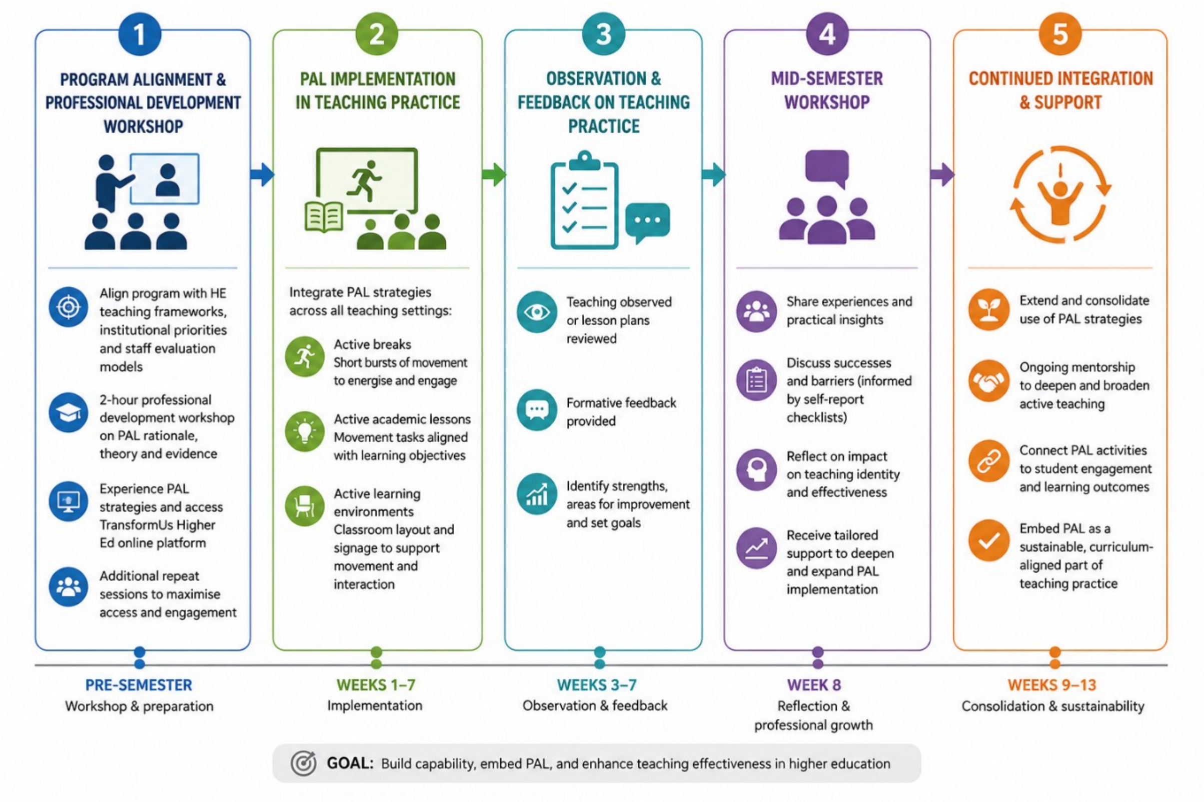
1
PROGRAM ALIGNMENT & PROFESSIONAL DEVELOPMENT WORKSHOP
Align program with HE teaching frameworks, institutional priorities and staff evaluation models
2-hour professional development workshop on PAL rationale, theory and evidence
Experience PAL strategies and access TransformUs Higher Ed online platform
Additional repeat sessions to maximise access and engagement
2
PAL IMPLEMENTATION IN TEACHING PRACTICE
Integrate PAL strategies across all teaching settings:
Active breaks Short bursts of movement to energise and engage
Active academic lessons Movement tasks aligned with learning objectives
Active learning environments Classroom layout and signage to support movement and interaction
3
OBSERVATION & FEEDBACK ON TEACHING PRACTICE
Teaching observed or lesson plans reviewed
Formative feedback provided
Identify strengths, areas for improvement and set goals
4
MID-SEMESTER WORKSHOP
Share experiences and practical insights
Discuss successes and barriers (informed by self-report checklists)
Reflect on impact on teaching identity and effectiveness
Receive tailored support to deepen and expand PAL implementation
5
CONTINUED INTEGRATION & SUPPORT
Extend and consolidate use of PAL strategies
Ongoing mentorship to deepen and broaden active teaching
Connect PAL activities to student engagement and learning outcomes
Embed PAL as a sustainable, curriculum-aligned part of teaching practice
PRE-SEMESTER
Workshop & preparation
WEEKS 1–7
Implementation
WEEKS 3–7
Observation & feedback
WEEK 8
Reflection & professional growth
WEEKS 9–13
Consolidation & sustainability
GOAL: Build capability, embed PAL, and enhance teaching effectiveness in higher education
