# Supplementary material for: Reimagining teaching in higher education: Impact of a pilot physically active learning intervention on perceived teaching effectiveness
Source: Front Sports Act Living. 2026 Sep 9;8:1942048. doi: 10.3389/fspor.2026.1942048 (PMC13597808; doi:10.3389/fspor.2026.1942048)
Supplement: Supplementary file 2 [file Supplementaryfile2.docx]

**Please indicate the degree to which you AGREE or DISAGREE with the following statements concerning YOUR FEELINGS about integrating physically active learning (PAL) into YOUR teaching:**

|  | **Strongly disagree** | **Somewhat disagree** | **Neither agree nor disagree** | **Somewhat agree** | **Strongly agree** |
| --- | --- | --- | --- | --- | --- |
| I would like to integrate physical activity into lectures and tutorials |  |  |  |  |  |
| I would enjoy integrating physical activity into lectures and tutorials |  |  |  |  |  |
| I feel apprehensive about integrating physical activity into lectures and tutorials |  |  |  |  |  |
| Integrating activity into lectures and tutorials may disrupt my teaching |  |  |  |  |  |
| Integrating activity into lectures and tutorials may enhance the curriculum |  |  |  |  |  |
| I would like to learn more about how to teach active lectures and tutorials |  |  |  |  |  |

**Please indicate the degree to which you AGREE or DISAGREE with the following statements concerning your feelings about physically active learning (PAL) on your STUDENTS OUTCOMES in your role as a LECTURER:**

|  | **Strongly disagree** | **Somewhat disagree** | **Neither agree nor disagree** | **Somewhat agree** | **Strongly agree** |
| --- | --- | --- | --- | --- | --- |
| I see increasing student physical activity as an important role for the lecturer |  |  |  |  |  |
| Integrating physical activity into lectures and tutorials would disrupt student learning |  |  |  |  |  |
| Integrating physical activity into class lessons would increase on-task behaviour of the students |  |  |  |  |  |
| Active breaks and active lessons would negatively affect academic outcomes |  |  |  |  |  |
| Integrating physical activity into lectures and tutorials would increase student focus |  |  |  |  |  |
| Integrating physical activity into lectures and tutorials would impede my ability to manage classroom behaviour |  |  |  |  |  |
| Activity lessons would increase student interest and engagement |  |  |  |  |  |

**For each aspect of physically active learning (PAL) listed in the table below, indicate how YOU perceive your LEVEL OF COMPETENCE:**

|  | **Very incompetent** | **Incompetent** | **Neither** | **Competent** | **Very competent** |
| --- | --- | --- | --- | --- | --- |
| Planning for PAL in lectures |  |  |  |  |  |
| Aligning PAL with unit and curriculum objectives |  |  |  |  |  |
| Delivering PAL as an instructional tool |  |  |  |  |  |
| Delivering PAL to engage students in learning |  |  |  |  |  |
| Aligning PAL with assessment objectives |  |  |  |  |  |
| Integrating PAL within assessments |  |  |  |  |  |
| Achieving desired unit outcomes |  |  |  |  |  |
| Managing the class |  |  |  |  |  |

**How CONFIDENT are you at...**

|  | **Not at all** | **A bit confident** | **Fairly confident** | **Quite confident** | **Very confident** |
| --- | --- | --- | --- | --- | --- |
| Planning for PAL in lectures |  |  |  |  |  |
| Aligning PAL with unit and curriculum objectives |  |  |  |  |  |
| Delivering PAL as an instructional tool |  |  |  |  |  |
| Delivering PAL to engage students in learning |  |  |  |  |  |
| Aligning PAL with assessment objectives |  |  |  |  |  |
| Integrating PAL within assessments |  |  |  |  |  |
| Achieving desired unit outcomes |  |  |  |  |  |
| Managing the class |  |  |  |  |  |

**Please indicate the DEGREE to which the following factors may act as BARRIERS OR INHIBIT the delivery of active lessons in your class**

|  | **No barrier or does not inhibit** | **Slight barrier or slightly inhibits** | **Moderate barrier or moderately inhibits** | **Significant barrier or strongly inhibits** | **Major barrier or extremely inhibits** |
| --- | --- | --- | --- | --- | --- |
| Inadequate training in PAL |  |  |  |  |  |
| Class size too big |  |  |  |  |  |
| Low levels of teaching confidence |  |  |  |  |  |
| Lack of time |  |  |  |  |  |
| Managing curriculum demands |  |  |  |  |  |
| Poor level of staff support provided |  |  |  |  |  |
| Inadequate facilities and equipment |  |  |  |  |  |
| Low levels of personal interest and enthusiasm |  |  |  |  |  |
| Poor personal experiences |  |  |  |  |  |
| Negative student attitudes towards PAL |  |  |  |  |  |
| Lack of assistance i.e. professional development or training opportunities |  |  |  |  |  |
| Litigation concerns |  |  |  |  |  |
| Teaching with learning difficulties |  |  |  |  |  |
| Teaching with behavioural challenges |  |  |  |  |  |
