# Supplementary material for: Reimagining teaching in higher education: Impact of a pilot physically active learning intervention on perceived teaching effectiveness
Source: Front Sports Act Living. 2026 Sep 9;8:1942048. doi: 10.3389/fspor.2026.1942048 (PMC13597808; doi:10.3389/fspor.2026.1942048)
Supplement: Supplementary file 3 [file Supplementaryfile3.docx]

**Please select the appropriate box to indicate how often you have incorporated the following into your teaching:**

|  | Never / No | Rarely | Sometimes | Often | As much as possible / Yes |
| --- | --- | --- | --- | --- | --- |
| **Planned** to deliver physically active learning in lectures and tutorials. |  |  |  |  |  |
| **Used** the physically active learning resources provided in planning. |  |  |  |  |  |
| **Integrated** physically active learning with in lectures and tutorials. |  |  |  |  |  |
| **Modelled** physically active teaching practices. |  |  |  |  |  |
| **Provided multiple opportunities** for students to be active in lectures and tutorials. |  |  |  |  |  |
| **Integrated** physically active learning within **assessment.** |  |  |  |  |  |
