# Supplementary material for: Reimagining teaching in higher education: Impact of a pilot physically active learning intervention on perceived teaching effectiveness
Source: Front Sports Act Living. 2026 Sep 9;8:1942048. doi: 10.3389/fspor.2026.1942048 (PMC13597808; doi:10.3389/fspor.2026.1942048)
Supplement: Supplementary file 4 [file Supplementaryfile4.docx]

**Semi-Structured Interview Guide**

**1. Prior knowledge and motivation**

- What did you know about physically active learning (PAL) before participating in this program?
- What motivated you to try PAL within your teaching?

**2. PAL implementation**

- What PAL strategies or activities did you use in your teaching?
- How frequently did you implement PAL during the semester?
- Can you describe an example of a lesson where you used PAL?

**3. Barriers to implementation**

- Were there any challenges to you delivering PAL?
- Were there any physical/social barriers?
- Were there any physical limitations (e.g., personal health) that impacted you or your students' ability to take part in the PAL strategies?

**4. Facilitators to implementation**

- What factors supported your implementation of PAL?
- Which organisational, collegial, or environmental supports were most helpful?

**5. Student influence**

- How did your students' responses to the PAL strategies make you feel?
- How did student engagement or feedback influence your implementation?

**6. Experiences with program resources**

Participants were shown the professional learning resources provided during the intervention (e.g., workshops, resource booklet, website, planning templates, activity guides, videos, observation and feedback, and teaching resources).

- Which resources did you use?
- Which resources did you find most useful?
- How did these resources support your implementation of PAL?

**7. Perceived impacts on teaching practice**

- How did you feel about embedding PAL into your teaching?
- Did the experience of using PAL energise or stress you? Can you share why you felt that way?
- Were there any habits or routines in your teaching that were hard to change?
- Have you noticed any changes in your teaching skills? How/In what way?
- Have you noticed any changes in your teaching confidence? How/Why?
- Has your knowledge, beliefs, and/or attitudes towards including PAL in your practice changed? What has changed/remained the same? Why do you think this happened?
- How has this program influenced your views about effective teaching practices?

**8. Perceived impacts on students**

- Do you think the PAL had an impact on your students? Can you share any examples that demonstrate the impact of PAL on your students?
- Is there any evidence that supported these observations (e.g., attendance, assessment outcomes, student feedback, teaching evaluations)?

**9. Overall reflections**

- What are your beliefs about the role of PAL in higher education?
- What student outcomes can PAL impact?
- What about for staff?
- Do you intend to continue using PAL in future teaching? Why or why not?

**10. Closing**

- Do you have any other information you would like to share, or have any other questions, comments or concerns?
